# Updated WHO nomenclature of head and neck lesions and associated imaging findings

**DOI:** 10.1186/s13244-019-0760-4

**Published:** 2019-07-16

**Authors:** Nisa Oren, Anatoliy Vaysberg, Daniel T. Ginat

**Affiliations:** Department of Radiology, Section of Neuroradiology, University of Chicago, 5841 S Maryland Avenue, Chicago, IL 60637 USA

**Keywords:** 2017 WHO nomenclature, Imaging, Tumors

## Abstract

This article reviews the imaging features of head and neck lesions with updated 2017 World Health Organization (WHO) nomenclature. The major WHO changes include refined terminology of existing entities, descriptions of new tumor types, elimination of defunct categories, and updated biological characterization of various tumor types. In particular, the updates pertaining to the following conditions will be reviewed: tumors of the oral cavity and oropharynx, including HPV-positive or HPV-negative squamous cell carcinoma, small cell carcinoma; tumors of the hypopharynx, larynx, trachea, and parapharyngeal space, including nomenclature revisions for laryngeal neuroendocrine tumors; tumors of the nasal cavity and paranasal sinuses including newly added entities such as NUT carcinoma and biphenotypic sinonasal sarcoma; odontogenic and maxillofacial bone tumors, including the reversal of terminology for certain cystic lesions; tumors of the salivary glands, including updated terminology related to high-grade transformation and polymorphous adenocarcinomas tumors; temporal bone lesions including modifications of the nomenclature and classification criteria; tumor-like lesions of the neck and lymph nodes, with a discussion encompassing developmental cysts, metastases of unknown primary, and heterotopia-associated neoplasia; and mucosal melanoma. Familiarity with the proper WHO terminology for conditions that might be mentioned in differential diagnoses and a general understanding of the behavior of head and neck lesions can help optimize imaging assessment and reporting.

## Key points

- There have been several recent changes to the WHO description of head and neck lesions.
- Changes include refinement of existing entities, description of new tumor types, elimination of defunct categories, and an update on the biology of various tumor types.
- Familiarity with the updated terminology and associated imaging findings is useful for optimally communicating relevant radiology exams.

## Introduction

The recently published 4th edition of the World Health Organization Classification of Head and Neck Tumors (WHO-HNT) has become an important reference the various disciplines related to otolaryngology. The major changes include refinement of existing entities, description of new lesions, elimination of defunct categories, and an update on the biology of various tumor types [1]. Familiarity with the latest terminology and corresponding imaging findings is important for accurately interpreting and communicating radiological findings pertaining to the lesions included in the updated WHO-HNT catalog. In particular, using the proper terminology for conditions that might be mentioned in differential diagnoses and understanding of the pathological behavior of the lesions can help optimize imaging assessment and reporting. Thus, the aim of this article is to review the updated 2017 WHO lesion nomenclature and associated imaging findings.

## Oral cavity and oropharynx tumors

**Fig. 1:**
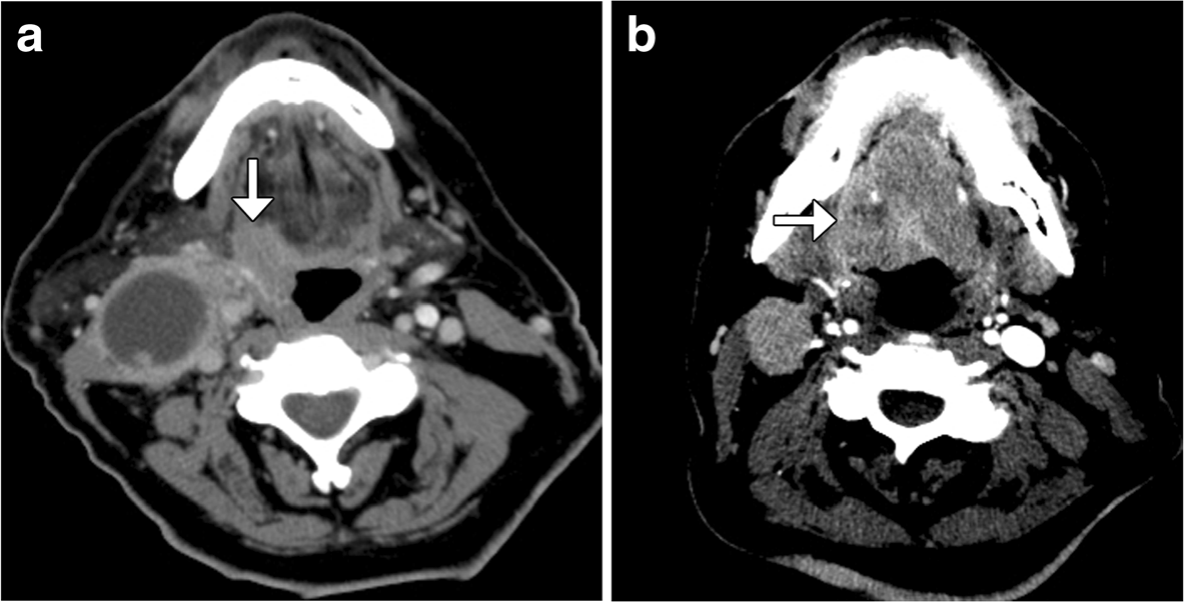
HPV-positive versus HPV-negative oropharyngeal squamous cell carcinoma. Axial CT image (**a**) shows a well-defined right oropharyngeal mass (arrow) and large cystic right cervical lymphadenopathy in a patient with HPV-positive squamous cell carcinoma. Axial CT image (**b**) shows an infiltrative right oropharyngeal mass (arrow) and solid right cervical lymphadenopathy in a patient with HPV-negative squamous cell carcinoma

One of the major changes in the new WHO-HNT is the recognition of oropharynx as a distinctive subsite [1, 2]. The other significant revision is classifying squamous cell carcinoma (SCC) of the oropharynx on the basis of human papilloma virus (HPV) status [2]. HPV-positive oropharyngeal cancers generally have more favorable prognosis than HPV-negative SCC [3]. There are certain imaging findings that may be helpful for differentiating HPV-positive SCC from the HPV-negative counterparts [4, 5]. In particular, HPV-positive SCC tend to display well-defined borders and cystic nodal metastases, while the HPV-negative tumors more commonly have poorly defined borders (Fig. 1) [4]. In addition, the metastatic lymph nodes associated with HPV-positive tumors tend to have lower apparent diffusion coefficient (ADC) values on diffusion weighted imaging (DWI) compared to the HPV-negative lymph node metastasis [5].

**Fig. 2:**
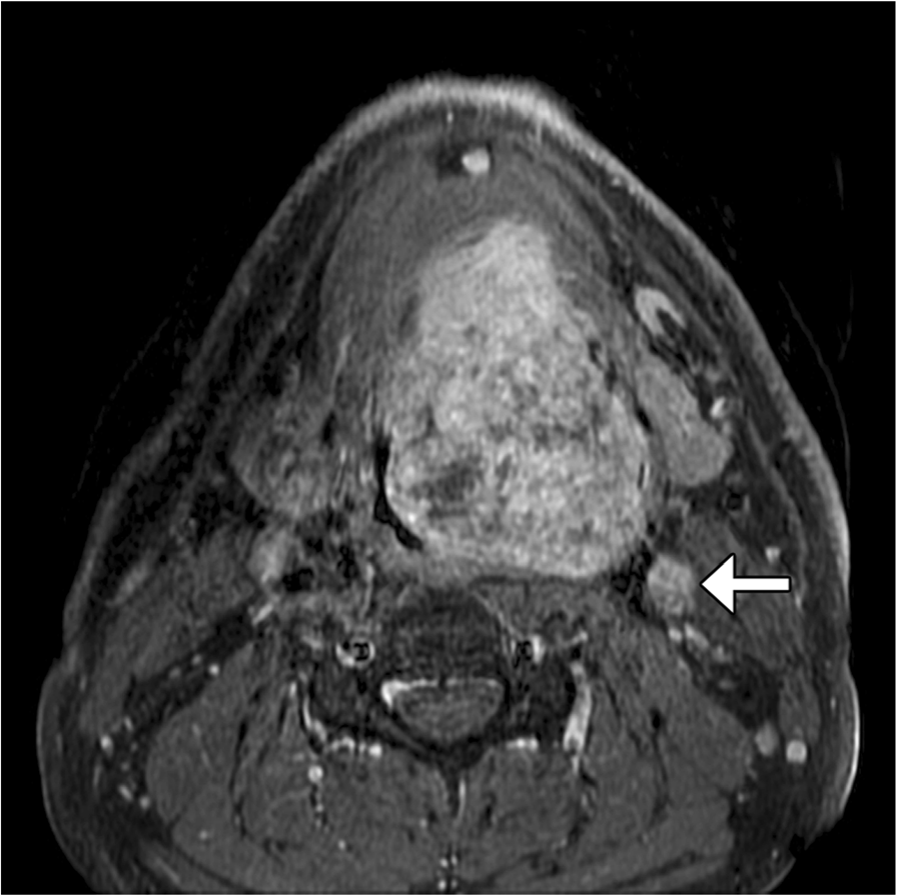
Polymorphous adenocarcinoma (PAC). Axial fat-suppressed T2-weighted MRI shows a bulky heterogeneous mass involving the oral cavity and tongue base with associated left level 2 metastatic lymphadenopathy (arrow)

The combination of polymorphous low-grade adenocarcinoma and cribriform adenocarcinoma of the tongue and minor salivary glands under a single-term polymorphous adenocarcinoma (PAC) is the other important difference between new and the old WHO-HNT [2]. This is a rare head and neck cancer, which generally has a good prognosis [6]. However, PAC has nonspecific imaging features, which include occasional adjacent bony invasion and erosion, but otherwise smooth borders with a fibrous capsule that appears as a hypointense rim on T2-weighted images, as well as a progressive enhancement pattern (Fig. 2). Rather than provide a specific diagnosis, the main responsibility of the radiologist is to assess the local extent of the tumor and to identify potentially metastatic lymph nodes [7].

## Hypopharynx, larynx, trachea, and parapharyngeal space tumors

WHO-HNT 2017 has significantly reduced the number of entities in the “Tumors of the Hypopharynx, Larynx, Trachea and Parapharyngeal Space” section, particularly in relation to tumors originating from the squamous epithelium. Laryngeal and hypopharyngeal conventional SCC, along with its variants and precursor lesions, comprise the majority of the chapter with emphasis on their etiological relationship with HPV infection. Careful analysis of the available data deemed laryngeal and hypopharyngeal verrucous SCC, spindle cell SCC, and basaloid SCC to be non-HPV-related tumors [8].

**Fig. 3.**
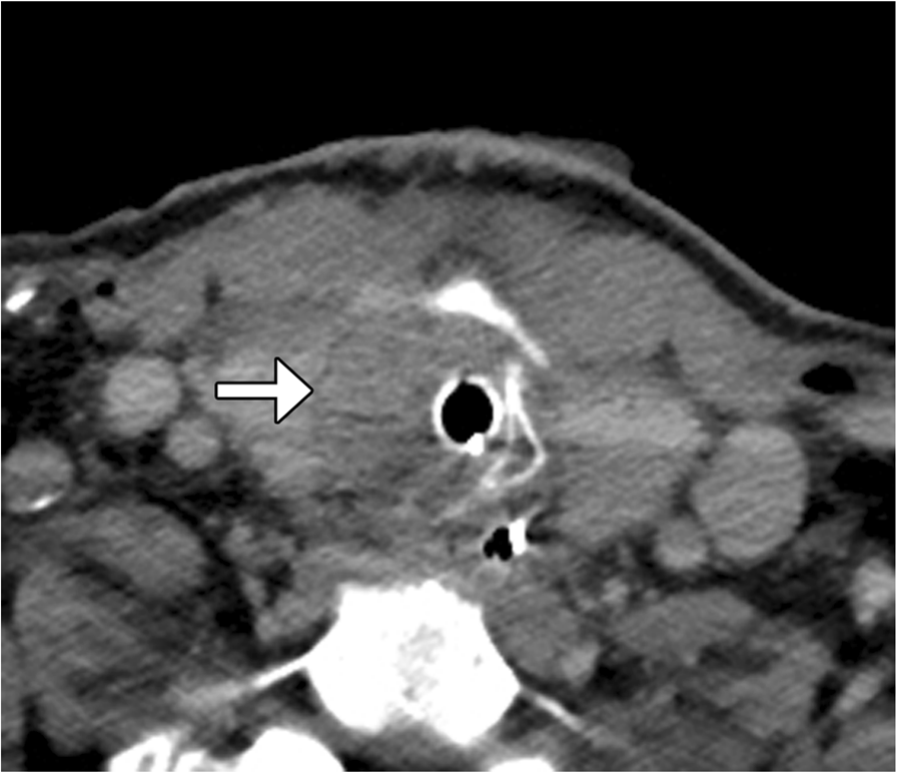
Laryngeal neuroendocrine carcinoma. Axial CT image shows an infiltrative right laryngeal mass with extralaryngeal extension (arrow)

In addition, the new WHO-HNT edition has changed the classification of laryngeal neuroendocrine carcinomas (NEC) as follows: well-differentiated NEC (carcinoid, grade I), moderately differentiated NEC (atypical carcinoid, grade II), and poorly differentiated NEC (grade III). The poorly differentiated NEC is furthermore separated into small cell NEC (SCNEC) and large cell NEC (LCNEC) [8]. The importance of transferring LCNEC into the grade III group of poorly differentiated NEC from the grade II atypical carcinoid/moderately differentiated neuroendocrine carcinoma category in the WHO-HNT 2005 edition is in signifying this entity’s specific morphology and associated poor prognosis [8]. NEC can appear as an infiltrative laryngeal mass on imaging, but the imaging findings are nonspecific and can resemble those of SCC (Fig. 3). Furthermore, there may be accompanying bulky metastatic cervical lymphadenopathy, typically without necrosis or cystic changes.

## Nasal cavity and paranasal sinus lesions

There are several new distinct entities in the nasal cavity and paranasal sinus regions that have been added to the latest edition of the WHO-HNT, including nuclear protein in testis carcinomas, human papillomavirus–related sinonasal carcinomas, SWI/SNF-related matrix-associated actin-dependent regulator of chromatin subfamily B member 1–deficient sinonasal carcinomas, renal cell-like adenocarcinomas, chondromesenchymal hamartomas, seromucinous hamartoma, NUT carcinoma, and biphenotypic sinonasal sarcoma (BSNS) [9].

**Fig. 4.**
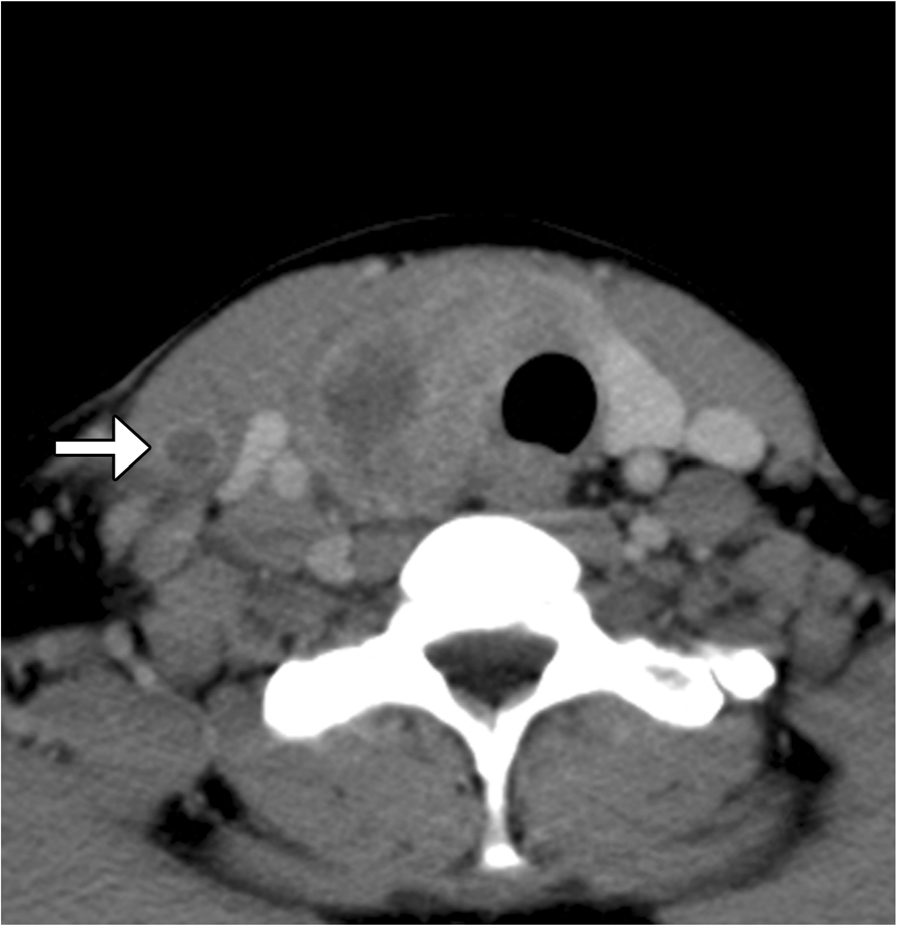
NUT carcinoma. Axial CT image shows an infiltrative mass in the right visceral space with involvement of the thyroid gland and necrotic right neck lymphadenopathy (arrow)

For example, NUT carcinoma, also known as NUT midline carcinoma, is a rare malignancy involving predominantly the midline structures of the body. The diagnosis is based on the presence of chromosomal rearrangements of the gene encoding nuclear protein of the testis at 15q14 with t(15;19) [10]. Although there is no defined particular anatomical site from which NUT carcinoma can arise, this entity has been included in this section given the fact that it commonly involves the sinonasal tract. However, this neoplasm can also arise from the upper airway, parotid gland, and even in the thyroid gland [11]. In addition, the tumor is not always strictly located in the midline. NUT carcinoma is characterized by its distinctively aggressive clinical course with a dismal median survival rate owing to a high incidence of regional and distant metastatic disease at the time of diagnosis and lack of effective treatment [12, 13]. On CT, NUT carcinoma has been described as a hypoattenuating infiltrative mass with heterogeneous enhancement with necrosis and poorly defined margins (Fig. 4). Otherwise, MRI can be useful in identifying bone marrow infiltration, perineural involvement, and skull base invasion [14].

**Fig. 5.**
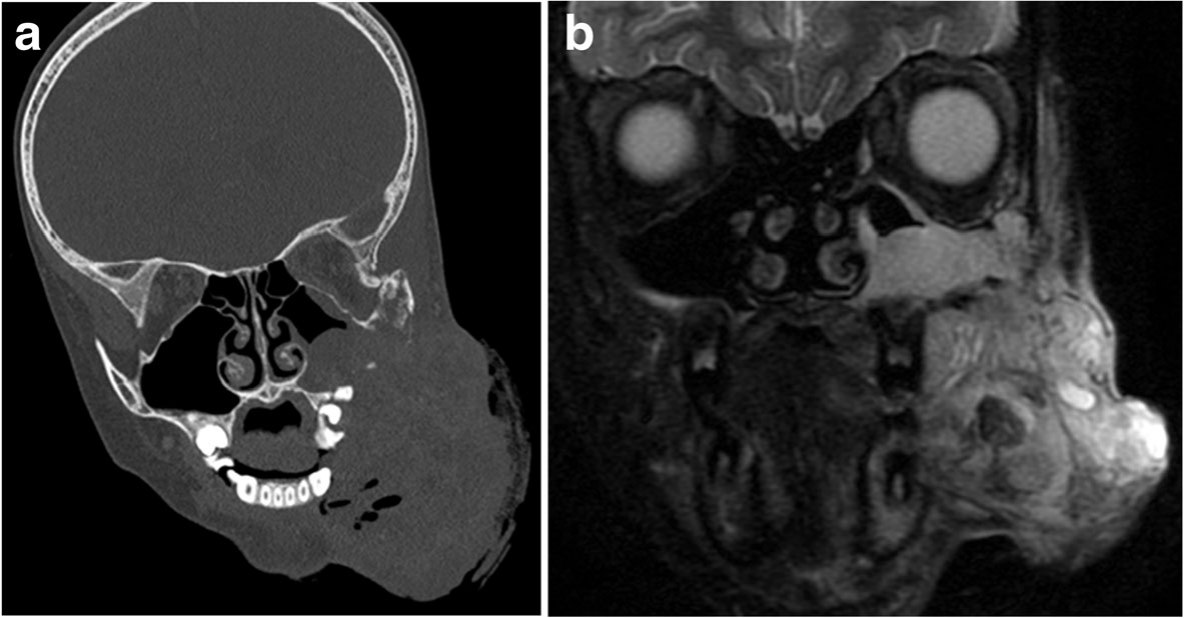
Biphenotypic sinonasal sarcoma. Coronal CT (**a**) and fat-suppressed T2-weighted MRI (**b**) show an aggressive bulky mass involving the left maxillary sinus and lower face

BSNS was initially termed low-grade sinonasal sarcoma with distinctive dual neural and myogenic features [15]. This rare tumor has a propensity to arise from the superior aspects of the nasal cavity and ethmoid sinuses, which can also lead to the adjacent orbital involvement [16]. There have been no reported cases of metastatic disease, although local recurrences have been frequently observed [16]. Association with hyperostosis of the adjacent bone has been described and is best assessed with CT, while areas of intratumoral cystic change or necrosis are particularly well delineated on MRI (Fig. 5) [17].

## Maxillofacial skeleton lesions

**Fig. 6.**
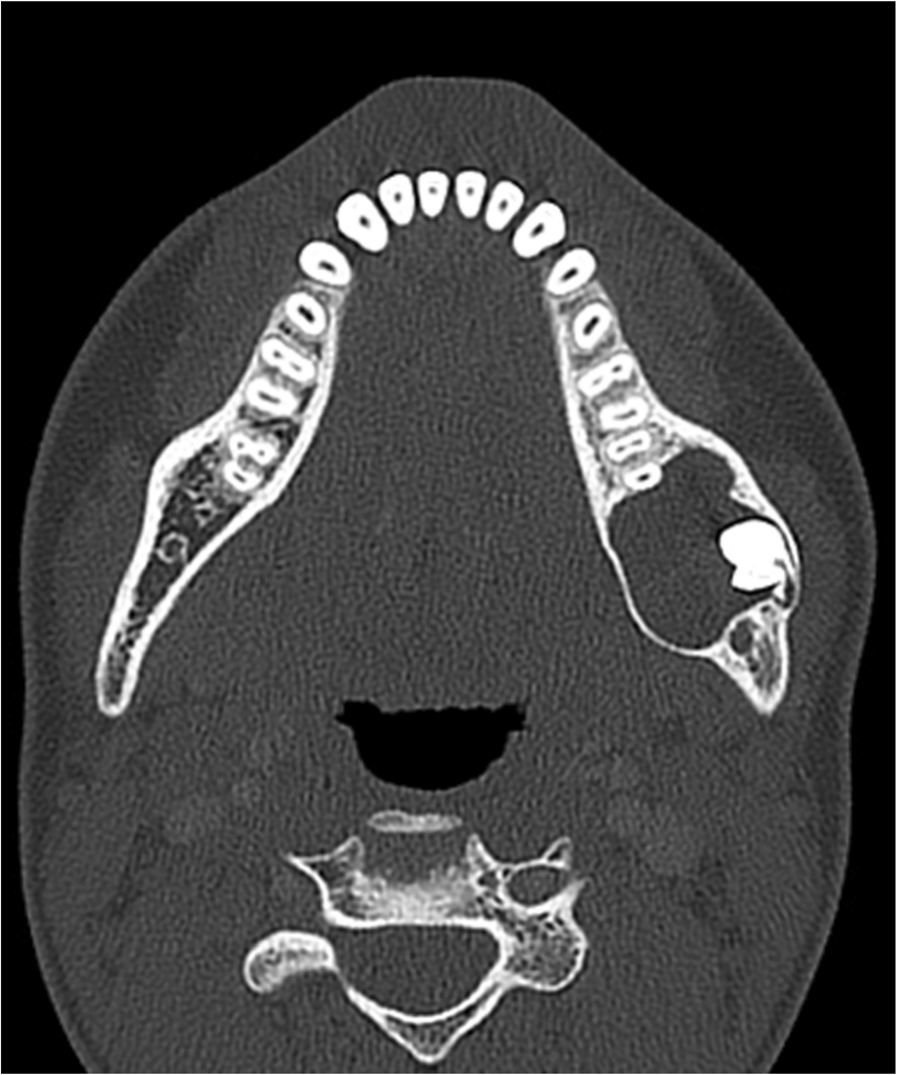
Odontogenic keratocyst. Axial CT image shows a well-defined expansile lucent lesion in the posterior left mandibular body surrounding the crown of an unerupted molar tooth

The emphasis of the new WHO-HNT classification is on discriminating between odontogenic tumors that are biologically benign versus those that are malignant [18]. While accurate, the 2005 edition was exceedingly complex and the 2017 version recognizes only epithelial, mesenchymal (ectomesenchymal), and mixed odontogenic tumors. One of the most debated topics in the 2017 classification was the decision to transfer keratocystic odontogenic tumor back into the cyst category as odontogenic keratocyst (OKC), with the evidence put forward for reclassification based on aggressive growth, high recurrence after treatment, and most importantly, mutations in the PTCH gene [19]. CT is considered the best imaging modality for this entity and the characteristic appearance is that of an expansile, solitary lucent lesion with smooth and often scalloped border rim, most commonly in the posterior mandible surrounding the crown of the third molar (Fig. 6). MRI will usually show a cystic lesion with or without thin peripheral enhancement, but no internal enhancement [20].

**Fig. 7.**
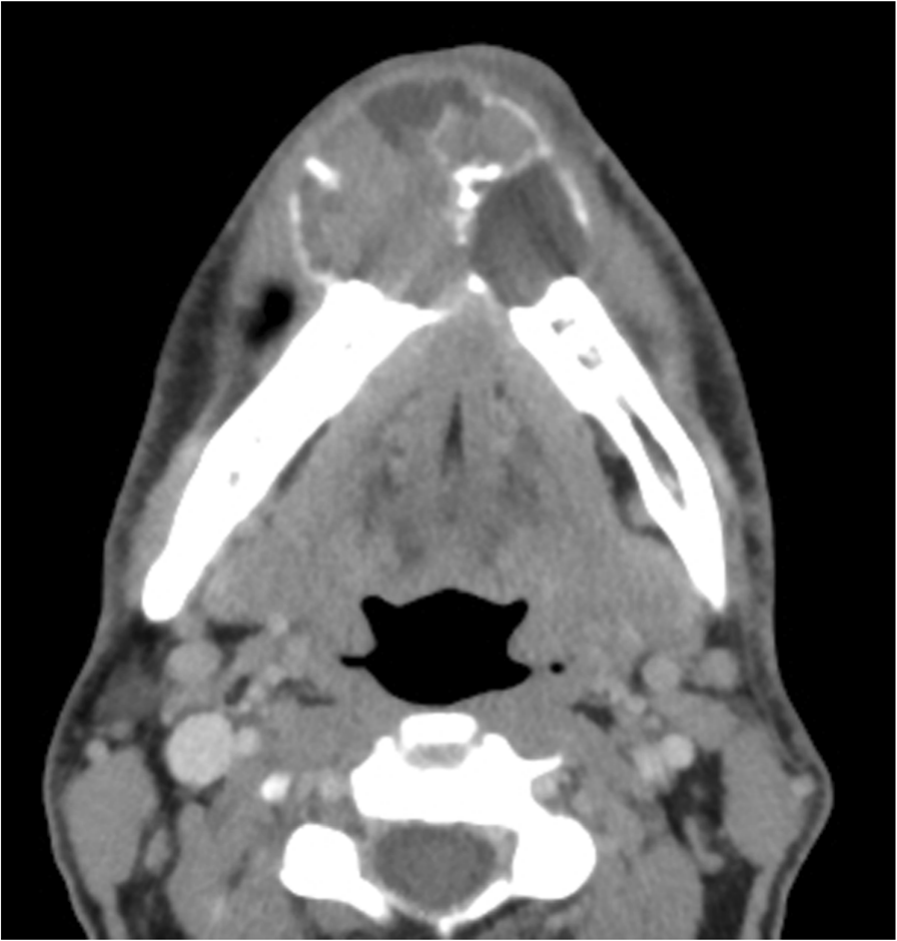
Ameloblastoma. Axial CT image shows a multilocular expansile lesion arising from the mandibular body

Regarding benign epithelial odontogenic tumors, the classification of ameloblastoma has been simplified to unicystic and extraosseous/peripheral types. A decision was made to remove the adjective “solid/multicystic” in reference to conventional ameloblastoma due to the lack of biologic significance. Despite their local aggressiveness and existence of an extremely rare variant known as metastasizing (malignant) ameloblastoma, ameloblastoma remained in the category of benign odontogenic epithelium tumors that represents approximately 10% of all odontogenic tumors, mostly diagnosed in young adults [19, 21]. CT features include a uni- or multilocular expansile lesion, commonly with “soap-bubble” pattern, scalloped borders, and extensive thinning of the cortex with larger lesions (Fig. 7). Resorption of adjacent teeth and unerupted molar tooth association are also common. Enhanced thin-section CT can delineate the relationship of the tumor to the bone and the presence of enhancing mural nodules [22, 23]. MRI is helpful in evaluating extraosseous components, including involvement of neurovascular structures and orbit. High T2 signal is typical of ameloblastoma on MRI, along with enhancement of septations and mural nodules, which can help to differentiate cases of cystic ameloblastomas from dentigerous cysts and odontogenic keratocysts [23]. However, the subtypes of ameloblastomas cannot be distinguished via radiology alone.

Ameloblastic carcinoma is a rare malignant lesion, which is more aggressive than the typical ameloblastoma, and can present with cortical bone destruction, extension into adjacent soft tissues, and a high rate of local recurrence and metastasis, particularly to cervical lymph nodes. The 2005 classification divided ameloblastic carcinoma into primary and secondary intraosseous tumors and secondary peripheral tumors. However, there is little justification to divide this very rare tumor into three types and is now simply defined as a single entity [19].

**Fig. 8:**
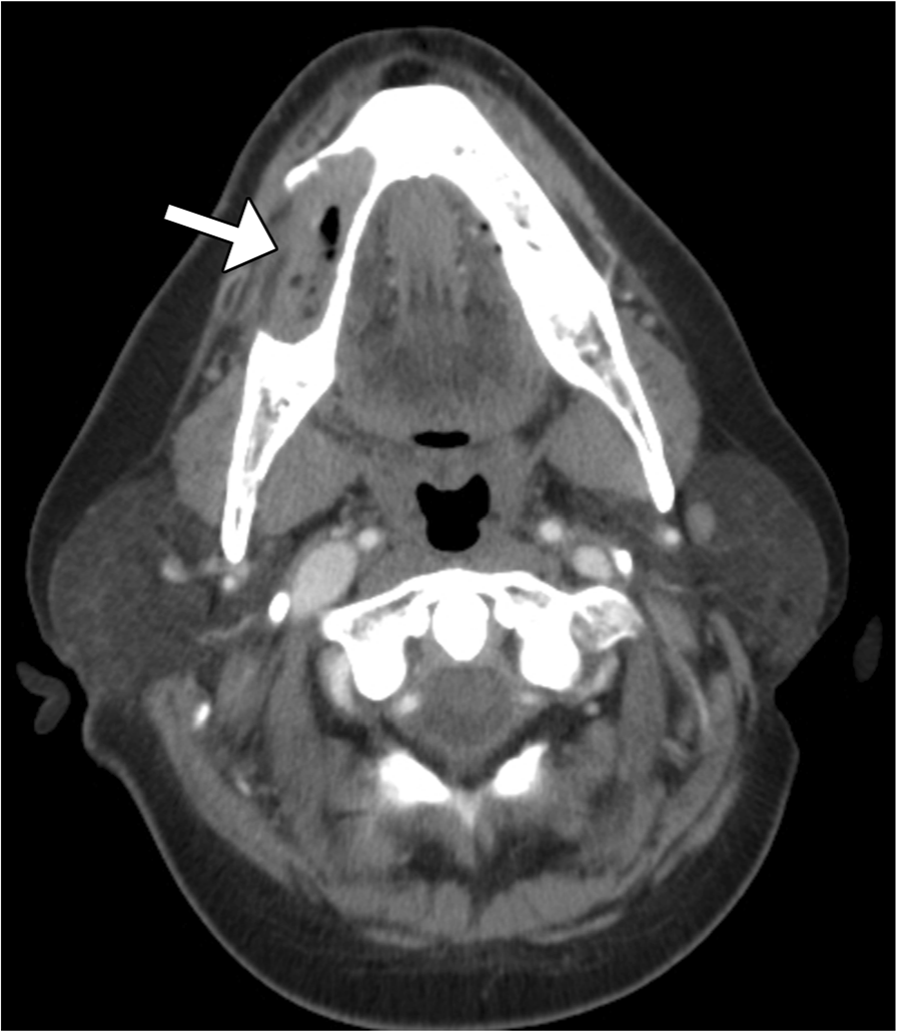
Ameloblastic carcinoma. Axial CT image shows a lytic lesion involving the right mandible (arrow)

Ameloblastic carcinoma and ameloblastoma can appear similar radiographically. However, imaging features such as erosion through the cortex with extension into the surrounding oral mucosa, extensive solid components, mixed solid and cystic components, and the occasional presence of focal dystrophic calcifications are more closely associated with ameloblastic carcinoma (Fig. 8) [24].

There were no new entities introduced to non-odontogenic maxillofacial bone tumors. However, as the understanding of genetic alteration of many neoplasms has evolved since 2005, most of the lesion descriptions were updated. For example, IDH1/2 mutations have been confirmed in approximately 50% of the cases of chondrosarcomas [19].

## Salivary gland tumors

Secretory carcinoma (SC), which has often been diagnosed as acinic cell carcinoma in the past, is a new addition to the 4th edition of WHO-HNT classification [25]. Although salivary secretory carcinomas can appear as solid enhancing masses, these tumors tend to contain large cystic components with an enhancing mural nodule and variable T2 signal on MRI [26–28]. In addition, a new category in the latest version of WHO-HNT is “other epithelial lesions,” which includes tumor-like lesions such as sclerosing polycystic adenosis (SPA).

**Fig. 9:**
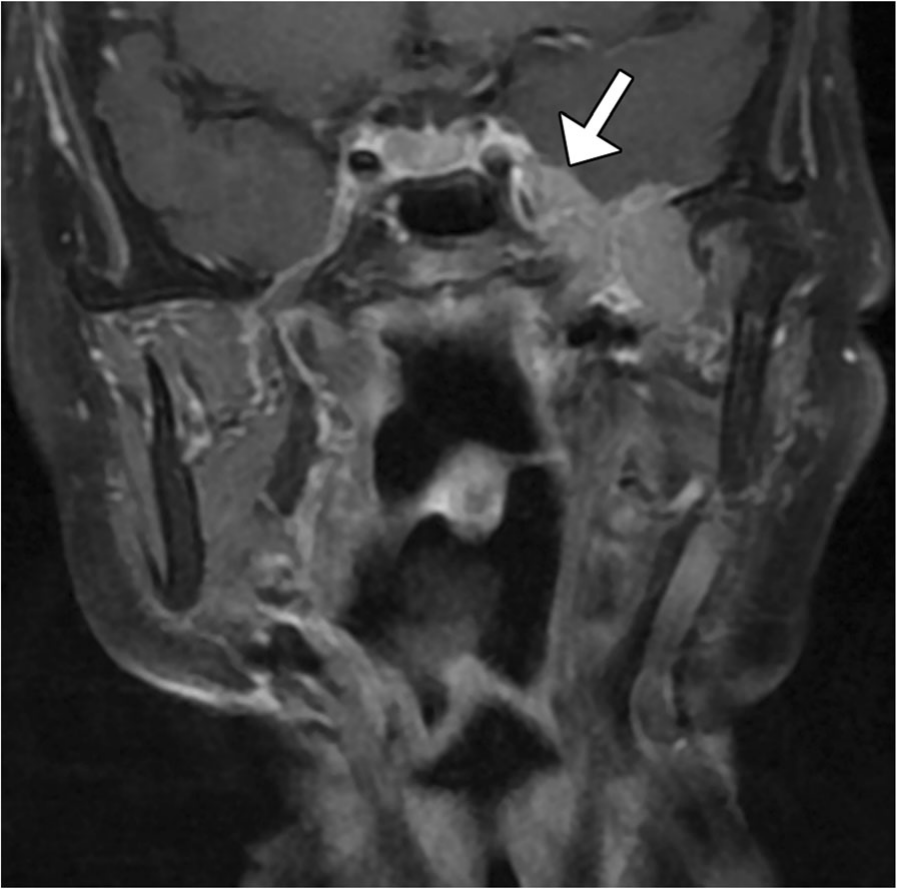
High-grade transformation of polymorphous low-grade adenocarcinoma originating from the parotid gland with perineural spread. Coronal fat-suppressed post-contrast T1-weighted MRI shows a mass in the left intratemporal fossa that extends through a widened foramen ovale into the left cavernous sinus (arrow)

High-grade transformation is now the preferred terminology in the new version of WHO-HNT over dedifferentiation for progression of a usually lower grade carcinoma with conventional morphology into a pleomorphic high-grade carcinoma such as acinic cell carcinoma, adenoid cystic carcinoma, and epithelial-myoepithelial carcinoma [25]. Perineural tumor extension is an important manifestation of salivary neoplasm with high-grade transformation to consider and this is best depicted via MRI. Perineural spread typically appears as enlargement and abnormal enhancement of the affected nerve and widening or obliteration of the nerve canal (Fig. 9).

## Temporal bone lesions

The 2017 WHO-HNT made several changes related to lesions involving the temporal bone. For example, the number of entities has been reduced by excluding lesions that do not occur solely or mostly at the site of the temporal bone, such as embryonal rhabdomyosarcoma, osteoma, exostosis, angiolymphoid hyperplasia with eosinophilia, Kimura disease, fibrous dysplasia, cholesterol granuloma, Schneiderian-type papilloma, inverted papilloma, choristoma, Paget disease of bone, neurofibromatosis 2, lipoma of the internal auditory canal, hemangioma, hematolymphoid tumors, and Langerhans cell histiocytosis. On the other hand, new entries include otosclerosis and cholesteatoma. Furthermore, modifications of the nomenclature and classification criteria of epithelial and ceruminous gland tumors of the middle and inner ear were made and the middle and inner ear were combined due to the limitations of imaging to delineate to precise site of origin [29]. Thus, temporal bone region neoplasms are classified anatomically as external auditory canal versus middle and inner ear tumors.

**Fig. 10:**
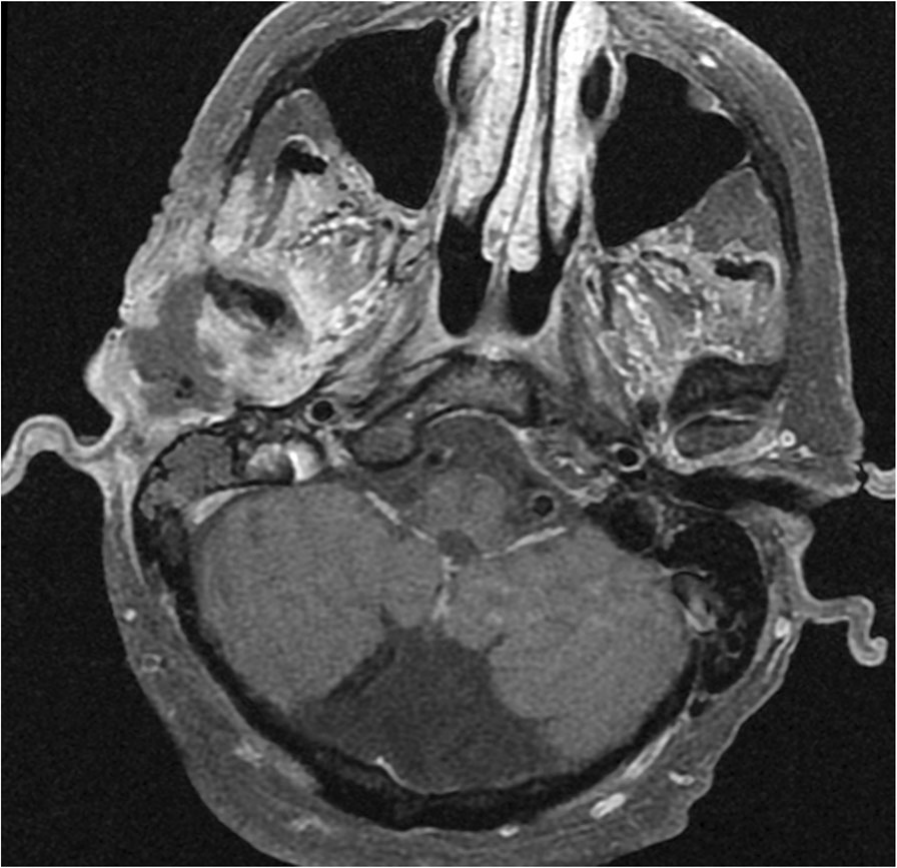
External auditory canal squamous cell carcinoma. Axial fat-suppressed post-contrast T1-weighted MRI shows an infiltrative and necrotic mass centered in the right external auditory canal with extension into the right temporomandibular joint space

The external auditory canal neoplasms in the 2017 WHO-HNT include squamous cell carcinoma, which is now listed as distinct from the external ear counterpart, and ceruminous tumors, including adenomas and adenocarcinomas [29]. On imaging, these lesions may appear as nonspecific soft tissue masses, but the role of imaging is to assess for potential invasion into adjacent tissues, including bone, which is relevant for treatment planning (Fig. 10).

**Fig. 11:**
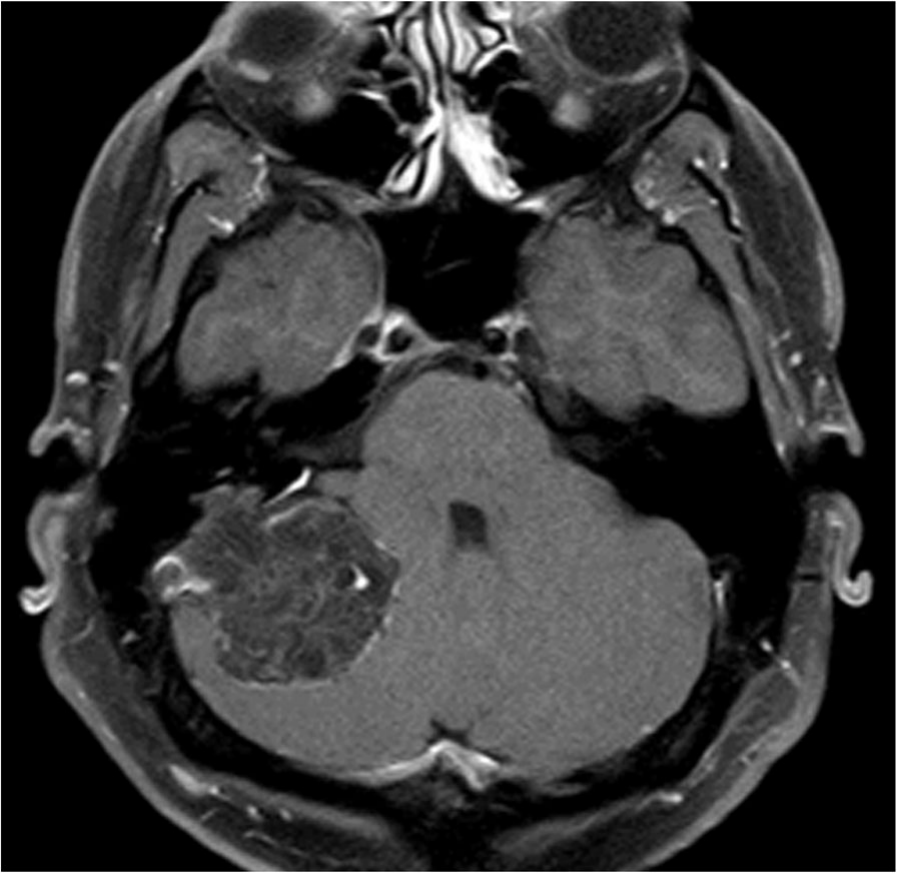
Endolymphatic sac tumor. Axial post-contrast T1-weighted MRI shows a bulky mass with mild heterogeneous enhancement that projects into the right posterior fossa from the posterior aspect of the right petrous bone

The middle and inner ear neoplasms that have updated descriptions in the updated 2017 WHO-HN include aggressive papillary tumor, which is an intermediate grade neoplasm, and endolymphatic sac tumor, which is a low-grade papillary epithelial neoplasm [29]. In particular, the association with von Hippel Lindau syndrome and the presence of certain imaging features, such as a bulky mass centered over the endolymphatic sac with irregularity of the posterior petrous bone, calcifications, and heterogeneous enhancement, can suggest the diagnosis of endolymphatic sac tumor (Fig. 11) [30].

## Tumors and tumor-like lesions of the neck and lymph nodes

**Fig. 12:**
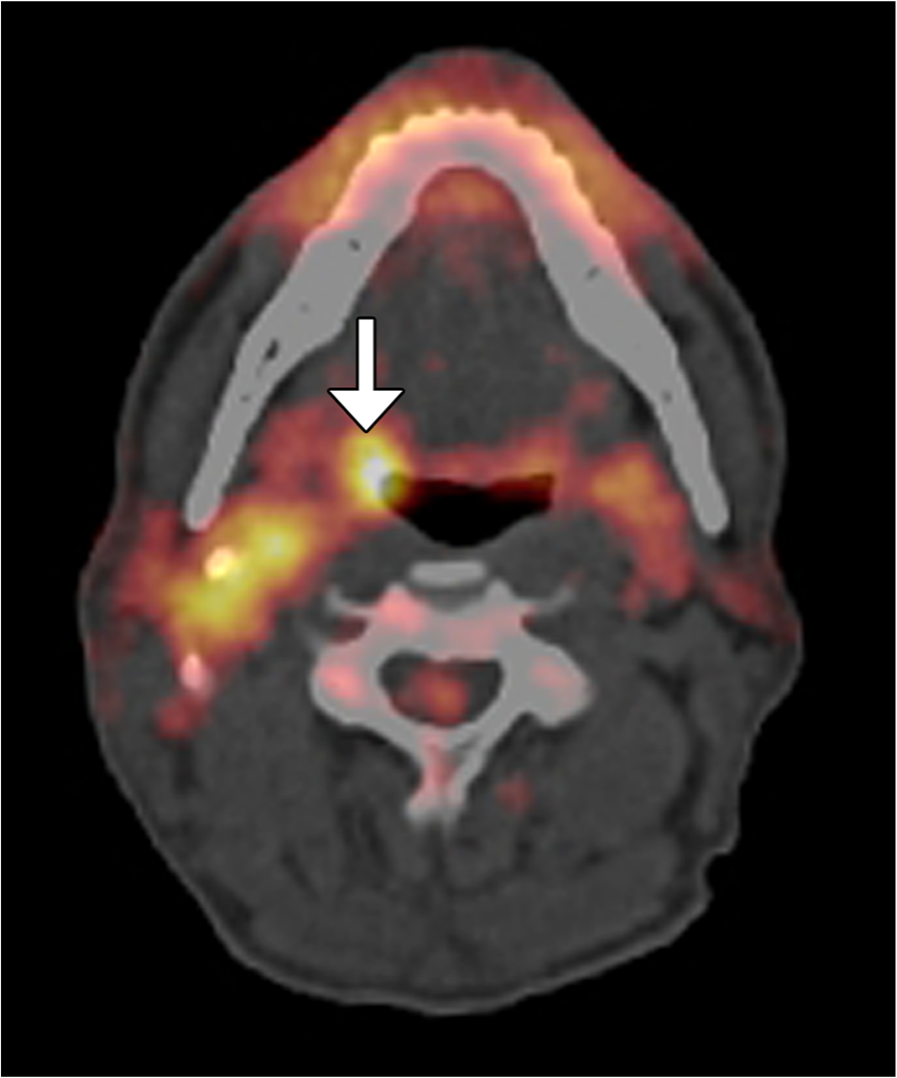
Tumor of unknown origin. ^18^FDG-PET/CT image shows right hypermetabolic cervical lymphadenopathy and a small ipsilateral hypermetabolic oropharyngeal lesion that proved to be the primary squamous cell carcinoma, which was not initially found on endoscopy

WHO-HNT 2017 now includes a section dedicated to tumors and tumor-like lesions of the neck and lymph nodes, including metastases. Some metastases have been given a diagnosis of unknown primary, but many are now identified as nasopharyngeal and oropharyngeal carcinomas that are often linked to EBV and HPV, respectively [36]. In such cases, radiologic assessment, including CT, MRI, and/or PET (Fig. 12), is mainly reserved for cases that are persistently negative despite endoscopic evaluation [31]. It is important not to dismiss cystic neck masses in young adults as congenital cysts, but to consider metastatic thyroid carcinoma or HPV-positive SCC in the differential diagnosis [32].

**Fig. 13:**
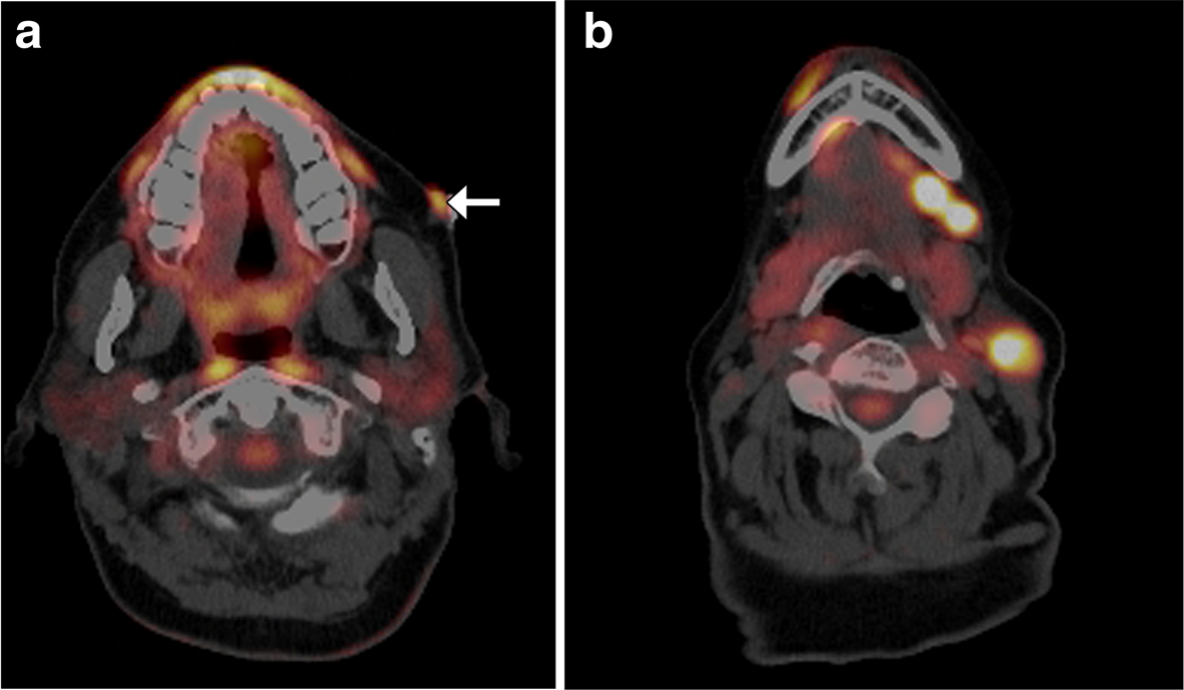
Merkel cell carcinoma. Axial fused ^18^FDG-PET/CT images (**a** and **b**) show a hypermetabolic left cheek lesion (arrow) and ipsilateral suprahyoid metastatic lymphadenopathy

The new WHO-HNT section on tumors and tumor-like lesions of the neck and lymph nodes also includes Merkel cell carcinoma (MCC). MCC is an aggressive cutaneous neuroendocrine tumor with a high propensity for lymph node and distal metastases that belongs to the family of small round blue cell tumors [33]. Although MCC involving the cervical lymph nodes almost certainly represents metastases from a primary skin malignancy, it is conceivable that there may be a primary nodal form of MCC. Regardless, since MCC tends to be avidly hypermetabolic, ^18^FDG PET/CT is the main modality used for staging (Fig. 13).

**Fig. 14:**
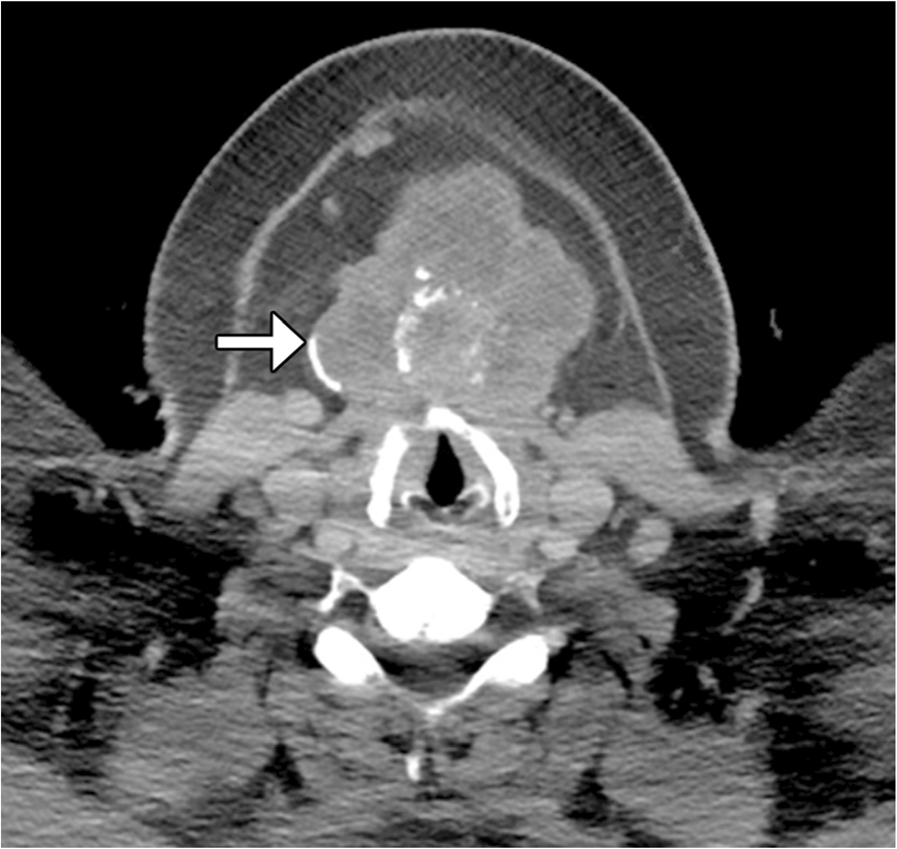
Papillary thyroid carcinoma arising from thyroglossal duct cyst. Axial CT image shows a bulky cystic and partly calcified mass in the midline anterior neck (arrow)

As opposed to metastases, heterotopia-associated carcinomas of the head and neck most commonly arise from ectopic salivary or thyroid tissue. For example, papillary thyroid carcinoma can arise from thyroglossal duct cysts (Fig. 14) [34]. The ectopic tumors can display analogous imaging features to their counterparts in regular locations. However, imaging may not necessarily help differentiate heterotopic primary tumors from lymph node metastases, which is an important consideration and may require detailed histological examination in order to make such a distinction.

**Fig. 15:**
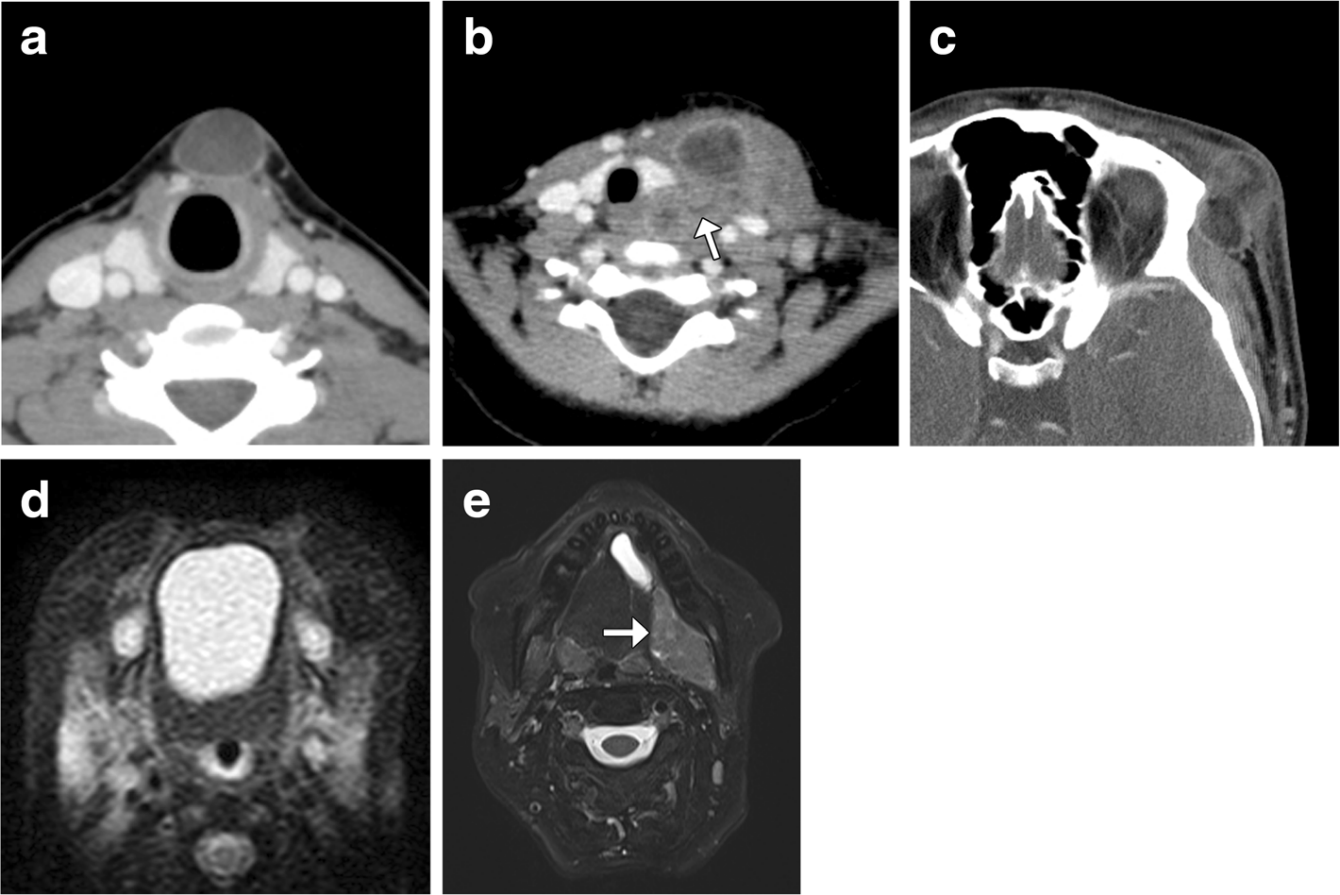
Various tumor-like lesions of the head and neck. Axial CT image (**a**) shows a midline anterior neck thyroglossal duct cyst. Axial CT image (**b**) shows an infected left neck branchial cleft cyst with surrounding inflammation and a distended sinus (arrow) that extends towards the hypopharynx. Axial CT image (**c**) shows a ruptured fat attenuation dermoid in the left lateral periorbital region with surrounding inflammation. Axial fat-suppressed T2-weighted MRI (**d**) shows a midline oral cavity foregut duplication cyst. Axial fat-suppressed T2-weighted MRI (**e**) shows a cystadenocarcinoma of the left sublingual region (arrow) associated with a ranula

There are many types of developmental and acquired cysts included in the updated WHO section on tumor-like lesions of the head and neck, including thyroglossal duct cysts, branchial cleft cysts, inclusion cysts, foregut duplication cysts, and ranulas (Fig. 15). While all of these can appear as simple cysts on imaging, the location of the lesions, such as midline versus lateral neck, can help distinguish them. Furthermore, some of the tumor-like lesions can display specific features, such as fat components in dermoids and sinus tracts with branchial cleft cysts. In addition, some of these cysts can be complicated by rupture and infection, which can manifest as cyst wall thickening and surrounding inflammation, and in rare cases, there may be an underlying neoplasm [35–38].

## Mucosal melanoma

**Fig. 16.**
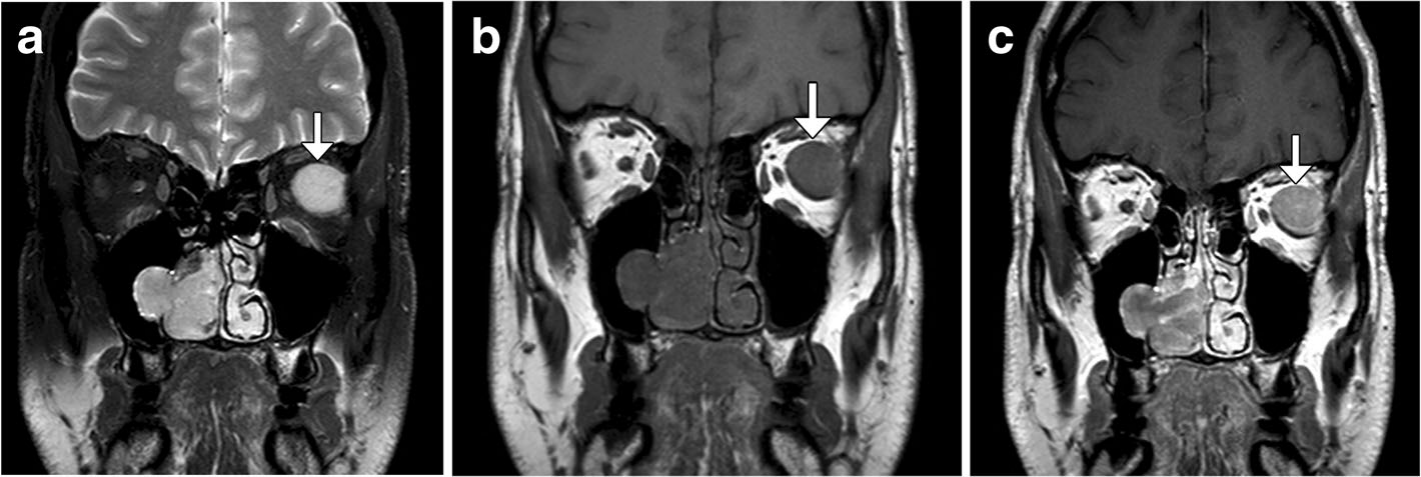
Metastatic mucosal melanoma. Coronal T2-weighted (**a**), T1-weigthed (**b**), and post-contrast T1-weighted (**c**) MR images show am enhancing high T2 and intermediate T1 signal right sinonasal mass with extension into the right maxillary sinus via a secondary ostium and a left lateral rectus metastasis (arrows)

The updated edition of WHO-HNT defines mucosal melanoma of the sinonasal and oral cavity as different subtypes. These are rare cancers of melanocytes, which are otherwise clinically and biologically distinct from cutaneous melanomas, with a worse overall prognosis due to a higher frequency of metastatic and multifocal disease [39, 40]. Mucosal melanoma appears nonspecific on CT and often consists of an enhancing soft tissue mass associated with adjacent osseous destruction. The MRI characteristics are governed by the chemical composition of the lesion, mainly the melanin content and presence of free radicals, metal ions, and methemoglobin. Melanotic melanomas classically exhibit high signal on T1-weighted sequences and variable T2 signal. On the other hand, amelanotic melanomas typically display low to intermediate T1 and high T2 signal. In either case, enhancement and invasion of surrounding spaces, as well as metastases can be demonstrated on MRI (Fig. 16).

## Data Availability

N/A

## Abbreviations

ADC: Apparent diffusion coefficient
CT: Computed tomography
DWI: Diffusion weighted imaging
FNA: Fine-needle aspiration
HNT: Head and neck tumors
HPV: Human papilloma virus
MCC: Merkel cell carcinoma
MR: Magnetic resonance
NCI: National Cancer Institute
NEC: Neuroendocrine carcinomas
OKC: Odontogenic keratocyst
PAC: Polymorphous adenocarcinoma
PTC: Papillary thyroid carcinoma
SCC: Squamous cell carcinoma
WHO: World Health Organization
